# Electropolymerization on ITO-Coated Glass Slides of a Series of π-Extended BODIPY Dyes with Redox-Active Meso-Substituents

**DOI:** 10.3390/molecules28248101

**Published:** 2023-12-15

**Authors:** Shawn Swavey, Alexa Wright

**Affiliations:** Department of Chemistry, University of Dayton, Dayton, OH 45469, USA; wrighta34@udayton.edu

**Keywords:** BODIPY, carbazole, electropolymerization, ITO

## Abstract

A series of meso-carbazole and meso-pyrene boron dipyrromethene(BDP) dyes have been synthesized using a two-step method. This simplified synthetic method did not require catalysts or oxidizing agents. Solution spectroscopic and electrochemical studies indicate that the HOMO and LUMO energies are dependent on the extent of π-conjugation associated with the pyrroles. Solution electrochemistry of the dyes in chloroform reveal film formation onto glassy carbon electrodes. Electrolysis of chloroform solutions of the dyes using indium tin oxide (ITO) glass slides as the working electrode show, using UV/vis spectroscopy, the formation of films. For two of the dyes, the BODIPY structure stays in tact upon electrolysis, exhibiting sharp absorption peaks on the ITO slides similar to that observed for the same dyes in solution.

## 1. Introduction

The fortuitous discovery of boron dipyrromethene (BDP) dyes by Triebs and Kruezer [1] in the late 1960s has, in the past two decades, received considerable attention, leading to tens of thousands of new BDP dyes. Molar absorptivities, often greater than 100,000 M^−1^cm^−1^, with near-unit quantum yields are two intrinsic properties that have contributed to the ever-growing interest in BDP dyes. Post-functionalization of BDP dyes has engendered bioactivity [2], catalytic properties [3], and has expanded their spectroscopic properties into the near-infrared (NIR) region of the electromagnetic spectrum [4]. Many BDP dyes are commercially available, primarily for use as fluorescent dyes for cellular organelles. Given their intense spectroscopic properties, these dyes have also been adapted for use as phototherapeutics [5] and, to a lesser extent, in dye-sensitized solar cells (DSSCs) [6].

**Scheme 1:**
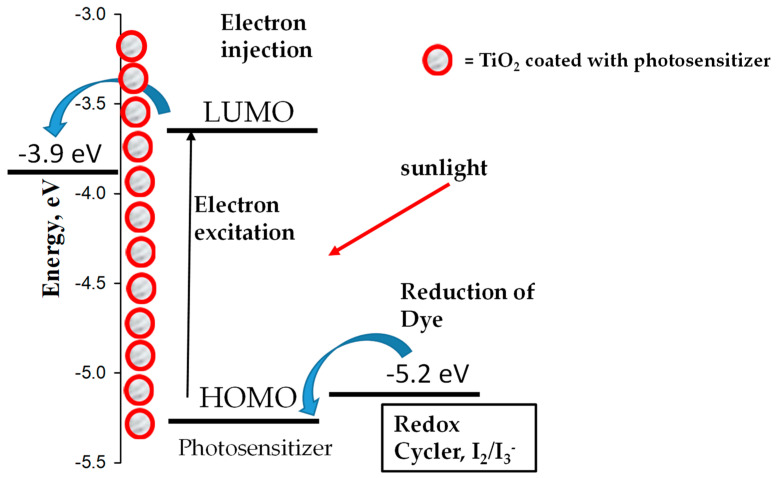
Electron collection at the anode of a DSSC.

DSSCs were first developed in the late 1980s, capturing a great deal of interest due to their relatively easy construction [7]. Many of the photosensitizers designed to work in DSSCs contain substituents like carboxylic acid groups to allow for covalent attachment and therefore increase solar conversion [8,9,10,11,12,13,14]. In principle TiO_2_ powder is coated with the photosensitizer, typically by adsorption, and the photosensitizer-TiO_2_ particles are placed on conducting transparent slides followed by heating prior to construction of the cell. Upon light irradiation, electrons from the highest occupied molecular orbital (HOMO) of the photosensitizer are transferred into the lowest energy molecular orbital (LUMO), leading to electron injection into the conduction band of the TiO_2_. Electron transfer from a redox cycler, for example, I_3_^−^/I^−^, regenerates the electronic structure of the dye (Scheme 1). One of the first reports of a BDP dye for DSSCs demonstrated the robust photostability of this class of dyes; however, low photocurrent production compared to known solar cell dyes was observed [15]. Subsequent studies have taken advantage of the post-functionalization diversity of BDP dyes to generate donor–acceptor photosensitizers for DSSCs [16,17].

**Figure 1:**
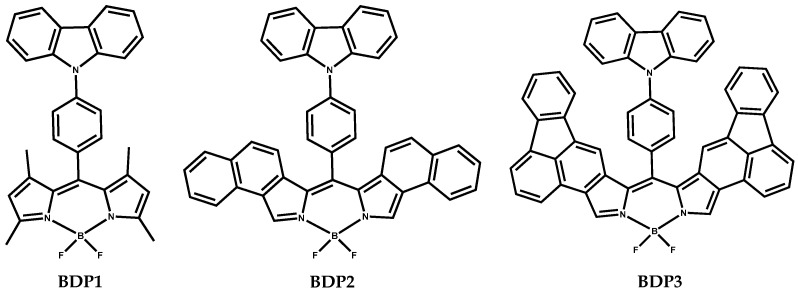
Proposed structures of **BDP1**, **BDP2**, and **BDP3**.

A number of examples of the electropolymerization of photosensitizers on indium tin oxide (ITO) glass slides have been reported [18,19,20,21]. This method has been shown to generate a more stable ITO film compared to spin-coating with the hope of generating efficient solar cells. To our knowledge, this technique has not been applied to BDP dyes. This report describes the straightforward synthesis of three meso-phenyl carbazole BDP dyes with varying degrees of pyrrole conjugation, Figure 1. Their solution phase spectroscopic and electrochemical properties are described. The ability of the dyes to electropolymerize onto ITO-coated glass slides is investigated by UV/vis spectroscopy. A fourth isoquinol–pyrene BDP dye is also investigated for its ability to electropolymerize onto ITO glass slides.

## 2. Results and Discussion

### 2.1. Synthesis and Spectroscopy

**Scheme 2:**
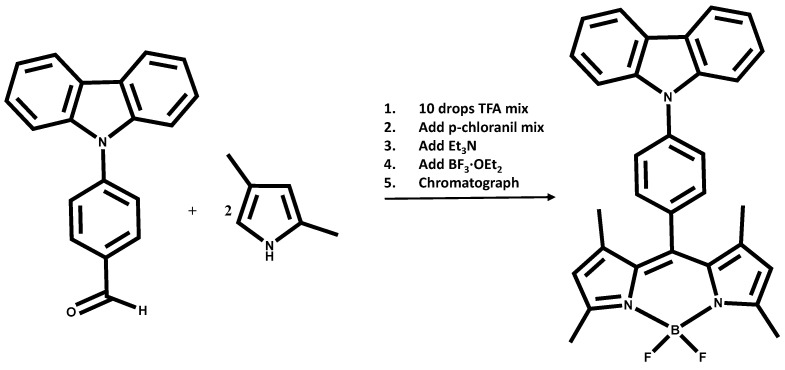
Synthesis of **BDP1**.

For the synthesis of **BDP1**, a mechanochemical technique [22] was used in which the commercially available N-(4-formylphenyl) carbazole and 2,4-dimethyl pyrrole were combined and mixed using a mortar and pestle with the addition of a few drops of trifluoroacetic acid followed by the addition of the oxidizing agent of p-chloranil. After 1 min of mixing, triethylamine followed by boron trifuoride etherate were added with additional grinding for 1 min. Chromatography of the reaction mixture yielded **BDP1** with a 28% yield (Scheme 2). This reaction methodology requires about 10 min to complete with yields similar to that achieved by longer, more traditional methods.

**Scheme 3:**
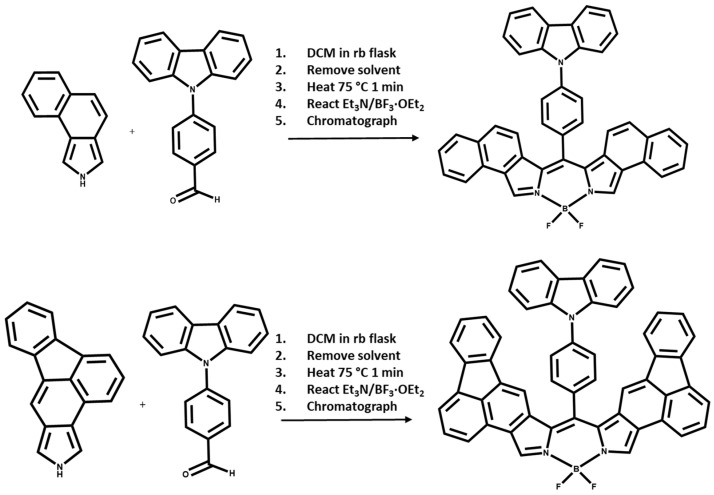
Synthetic scheme for **BDP2** and **BDP3**.

This laboratory has described a two-step synthetic route for BDP dyes containing highly conjugated pyrroles [23]. This solvent-free reaction does not require an oxidizing agent to produce the dipyrrin and can be completed in approximately 30 min. The appropriate pyrrole (naphthyl pyrrole or fluoranthene pyrrole) with N-(4-formylphenyl) carbazole was placed in a round bottom flask and dissolved in a minimum of chloroform to make a homogeneous solution. The solvent was subsequently removed under reduced pressure with heating for 1 min, generating a deep purple/red paste. The flask containing the reaction mixture was placed in an oil bath set at 80 °C with a nitrogen purge while dry toluene was added followed by triethylamine and boron trifluoride etherate. **BDP2** and **BDP3** were isolated by column chromatography with yields of 16.5% and 10.0%, respectively, Scheme 3.

**Figure 2:**
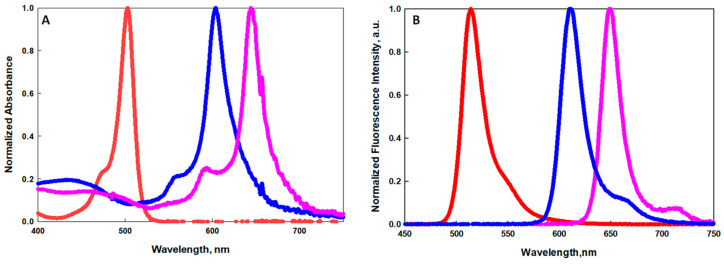
(**A**) Electronic spectra of **BDP1** (red), **BDP2** (blue), and **BDP3** (violet) at room temperature in chloroform. (**B**) Emission spectra **BDP1** (red), **BDP2** (blue), and **BDP3** (violet) excited at their respective absorption maxima in chloroform.

UV/vis spectroscopy of BDP dyes shows strong absorption in the visible region of the electromagnetic spectrum resulting from HOMO (π) to LUMO (π*) transitions localized on the dipyrrin core with BF_2_ serving to align the aromatic pyrrole units. The meso-substituent typically plays a limited role in the energy of the absorption since these substituents are orthogonal to the dipyrrin core. Therefore, shifting the absorption to lower energy often requires post-functionalization of the BDP dye, adding synthetic steps and decreasing the yield [24]. Our methodology allows for predictable bathochromic shifts in the spectrum as a function of the degree of conjugation of the pyrroles used. The electronic spectra of the carbazole-substituted BDP dyes show intense absorption peaks at 504 nm (**BDP1**, red), 604 nm (**BDP2**, blue), and 644 nm (**BDP3**, violet), consistent with the extent of their dipyrrin conjugation, Figure 2A. Molar absorptivities of the dyes range from 66,760 M^−1^cm^−1^, 55,480 M^−1^cm^−1^, and 43,340 M^−1^cm^−1^ for **BDP1**, **BDP2**, and **BDP3**, respectively. Excitation at their absorption maxima show sharp intense emission (Figure 2B) with peaks at 514 nm (**BDP1**, red), 611 nm (**BDP2**, blue), and 649 nm (**BDP3**, violet) with quantum yields of 0.48, 0.47, and 0.37, respectively.

### 2.2. Solution Cyclic Voltammetry

**Figure 3:**
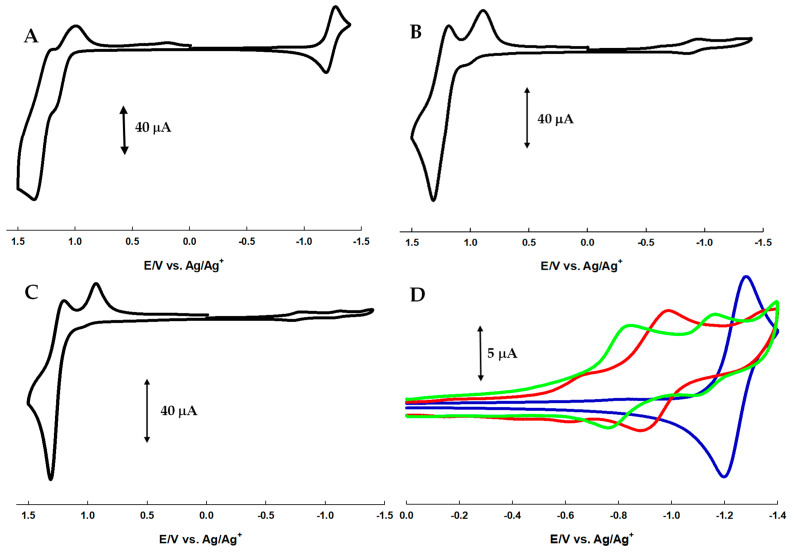
Cyclic voltammograms of mM chloroform solutions of **BDP1** (**A**), **BDP2** (**B**), and **BDP3** (**C**) containing 0.1 M TBAPF_6_ as supporting electrolyte. Glassy carbon working electrode, a platinum wire auxiliary, and non-aqueous Ag/Ag^+^ reference electrode. Scan rates of 100 mV/s. (**D**) overlay of cathodic region of (**A**–**C**) with blue (**BDP1**), red (**BDP2**), and green (**BDP3**).

Solution cyclic voltammetry was performed on chloroform solutions of the BDP dyes using a three-electrode one-compartment cell with a glassy carbon working electrode, platinum wire auxiliary electrode, and a non-aqueous Ag/Ag^+^ reference electrode. Scanning in the anodic direction, all three dyes display an intense oxidation wave with an E_pa_ of 1.35 V, 1.31 V, and 1.31 V vs. Ag/Ag^+^ for **BDP1**, **BDP2**, and **BDP3**, respectively. These oxidation waves are coupled to a weaker reduction wave at 0.99 V, 1.18 V, and 1.20 V vs. Ag/Ag^+^, Figure 3A–C. **BDP2** and **BDP3** show an additional reduction wave at 0.88 V and 0.92 V, respectively. In the cathodic direction, reversible redox waves with E_1/2_ values −1.24 V (ΔE 70 mV), −0.92 V (ΔE 60 mV), and −0.76 V (ΔE 70 mV) for **BDP1**, **BDP2**, and **BDP3**, respectively, which are highlighted in Figure 3D where **BDP1** (blue), **BDP2** (red), and **BDP3** (green). The one-electron reversible cathodic redox couple for **BDP1**, **BDP2** and **BDP3** shows a shift to less negative values, respectively, consistent with the increasing conjugation of the dyes; therefore, these redox couples are assigned as the reduction and oxidation of the LUMO localized on the dipyrrin core, in agreement with the spectroscopic data. The similarity of the irreversible oxidation waves for the three dyes is likely due to the oxidation of the meso-substituent carbazole.

**Figure 4:**
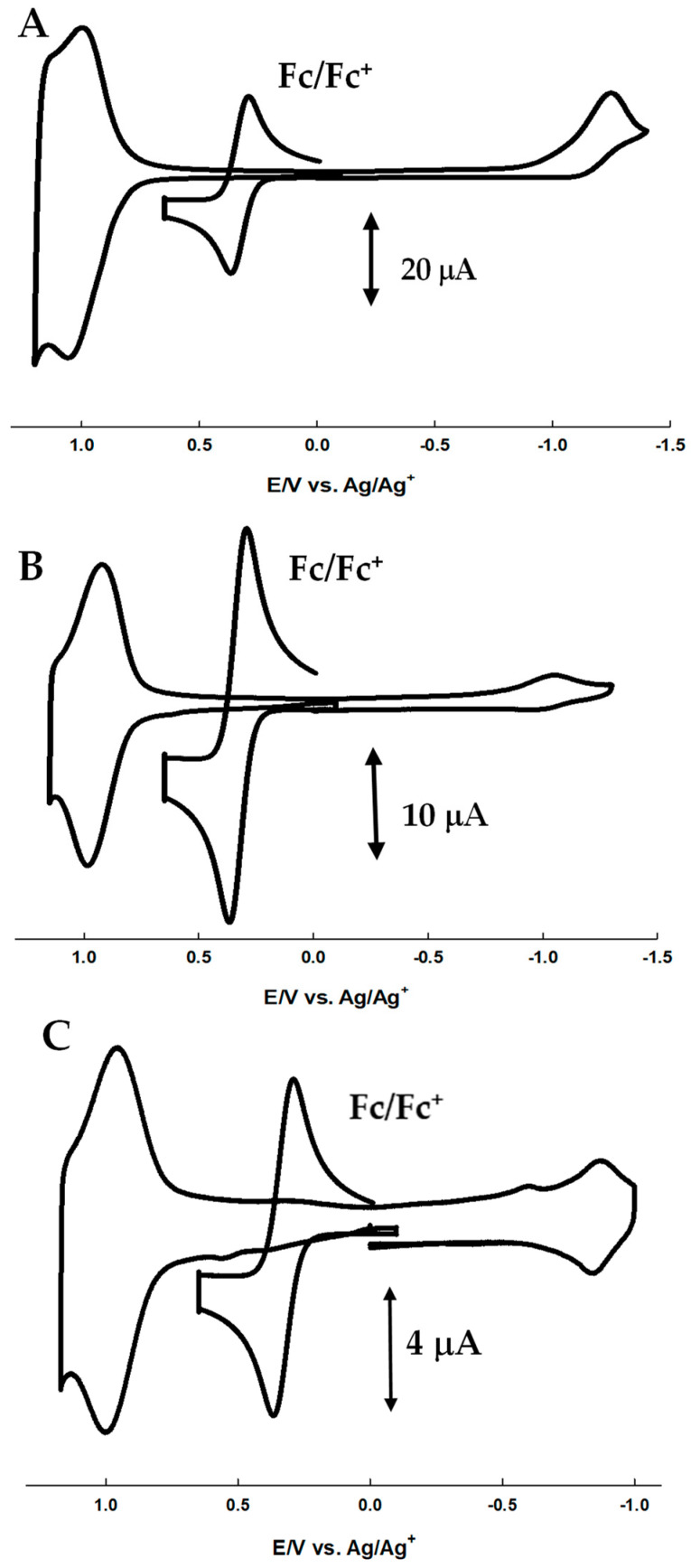
Cyclic voltammetry of glassy carbon electrodes with adsorbed **BDP1** (**A**), **BDP2** (**B**), and **BDP3** (**C**) in 0.1 M TBAPF_6_ chloroform solutions, with a platinum wire auxiliary electrode and a nonaqueous Ag/Ag^+^ reference electrode. Fc/Fc^+^ redox couple measured in under the same conditions is overlayed. Scan rates of 100 mV/s.

Scanning repeatedly in the anodic direction led to the formation of a film on the glassy carbon electrode resulting from electro-polymerization of the carbazole moieties [25]. Once the electro-oxidative film of **BDP1**, **BDP2**, and **BDP3** dyes was formed on the glassy carbon electrodes, they were removed and dipped in chloroform. The electrodes were then placed into chloroform solutions containing only supporting electrolyte (0.1 M TBAPF_6_) and cycled between 1.2 V and −1.5 V, Figure 3. The redox couples observed in the anodic region are indicative of adsorbed reversible electron transfer [26]. All three adsorbed dyes show anodic redox couples (Figure 4A–C) at 0.98 V vs. Ag/Ag^+^ with reduction waves at −1.26 V (**BDP1**), −1.06 V (**BDP2**), and −0.88 V (**BDP3**), consistent with increasing π-conjugation, respectively.

**Figure 5:**
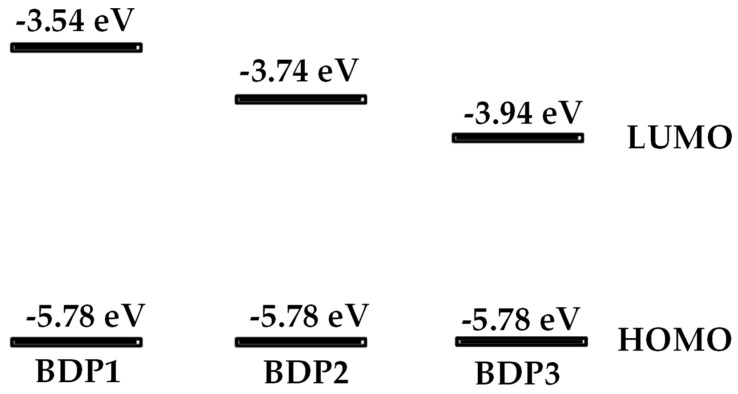
Qualitative Jablonski diagram of the HOMO/LUMO energies determined from cyclic voltammograms of the adsorbed BDP dyes from Figure 3.

The CVs illustrated in Figure 4 show an overlay of the ferrocene redox couple run under the same conditions. Using the Fc/Fc^+^ redox couple, the HOMO/LUMO energies associated with each of the dyes can be calculated [27], resulting in a qualitative Jablonski diagram, Figure 5. The HOMO orbitals are linked to the anodic redox couple resulting from the electrooxidative polymerization of the carbazole moieties common to all three dyes and therefore occurring at the same energies. The LUMO orbitals are localized on the dipyrrin core of the BDP dyes and their energies are determined from the cathodic reduction waves, Figure 4A–C. The associated energies of the LUMOs vary as a function of the increasing π-conjugation going from **BDP1** to **BDP3**.

**Scheme 4:**
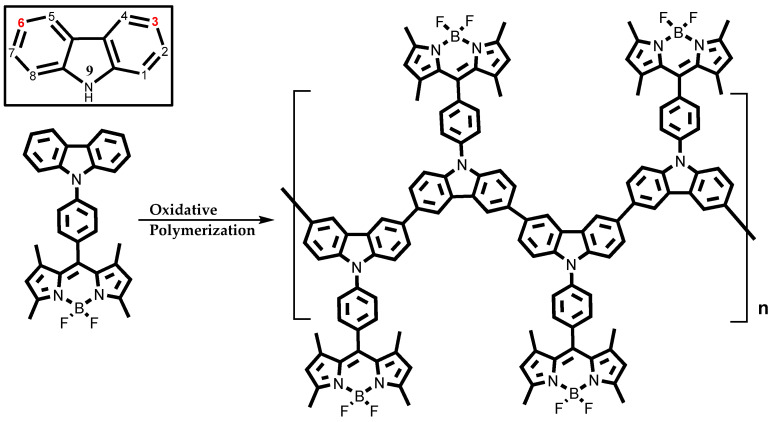
Proposed structure of electropolymerized **BDP1** on ITO glass slides.

Generation of polycarbazoles has been studied for several decades. Chemical polymerizations of carbazoles have been performed by using various oxidizing agents [28]. The electrochemical oxidation of carbazoles has been extensively studied due to their ability to form conductive films on platinum, glassy carbon, and ITO surfaces [29,30,31,32]. A vast array of applications for electrochemically generated conductive polycarbazole films has been noted [33,34,35,36,37,38,39,40,41,42,43,44]. While there exist a number of mechanisms for the electrochemical formation of polycarbazoles, it is generally agreed that an initial one-electron oxidation generates a radical with the 3,6-positions of the carbazole, representing the most reactive positions, inset-red Scheme 4, resulting in a chain reaction culminating in the formation of polycarbazole films [38]. Employing suggested polycarbazole film structures, Scheme 4 illustrates a proposed polycarbazole-BDP film formed via the oxidation of **BDP1** with a similar structure resulting from the oxidative electropolymerization of **BDP2** and **BDP3**.

### 2.3. ITO Film Formation of BDP1–BDP3

**Figure 6:**
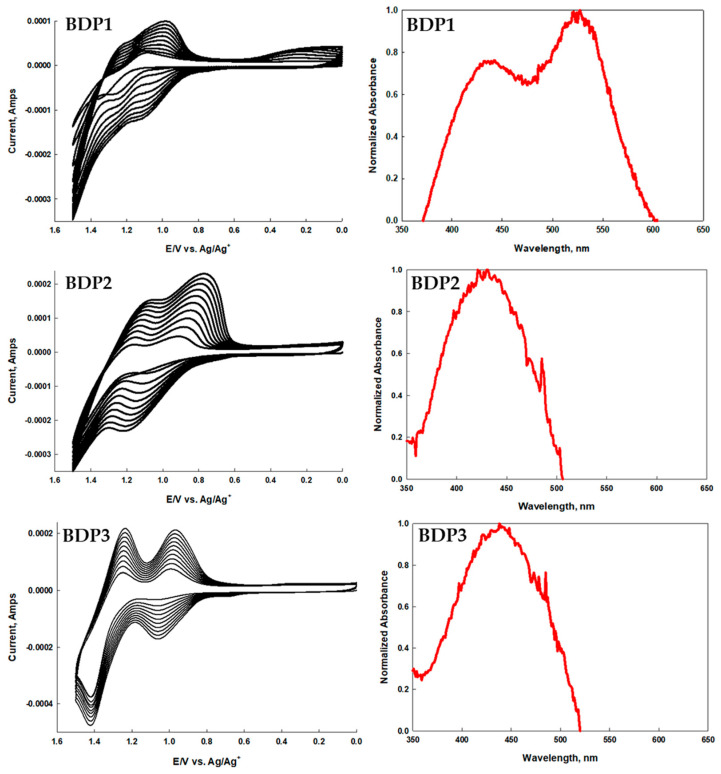
(**top**) Cyclic voltammetry of mM chloroform solutions of BDP dyes with 0.1 M TBAPF_6_ as supporting electrolyte, a square ITO glass slide as working electrode, platinum flag auxiliary electrode, and non-aqueous Ag/Ag^+^ reference electrode. (**bottom**) UV/vis spectra of ITO slides after electrolysis of the BDP dye solutions for 100 s at 1.5 V.

Square ITO-coated glass slides were placed in millimolar solutions of **BDP1**, **BDP2**, and **BDP3** containing TBAPF_6_ as a supporting electrolyte with a platinum flag auxiliary electrode and a nonaqueous Ag/Ag^+^ reference electrode. Figure 6, left, illustrates cyclic voltammograms of these solutions from 0 V to 1.5 V for 10 cycles. The CV’s in Figure 6 with square ITO glass slides as the working electrode show the buildup of the BDP films. To obtain a detectable film on the ITO slides by UV/vis spectroscopy, bulk electrolysis of the mM solutions was performed at 1.5 V vs. Ag/Ag^+^ reference electrode for 100 s, at which point, the slides were rinsed with chloroform and air-dried. UV/vis spectra of the ITO-films for the respective dyes are illustrated in Figure 6 (right). The UV/vis spectrum of the ITO-**BDP1**-coated glass slide shows two broad absorption bands with peaks at 440 nm and 530 nm. The absorption spectra of ITO slides electrocoated with **BDP2** and **BDP3** show similar broad bands with peaks at 440 nm but do not show any additional absorption bands.

To better understand the discrepancies of the UV/vis spectra of the ITO-coated slides, chloroform solutions of N-(4-formylphenyl) carbazole were bulk-electrolyzed at 2.0 V vs. a nonaqueous Ag/Ag^+^ reference electrode utilizing an ITO glass slide as the working electrode. After 100 s of electrolysis, the ITO glass slide was removed from the solution, thoroughly rinsed with chloroform and air-dried.

**Figure 7:**
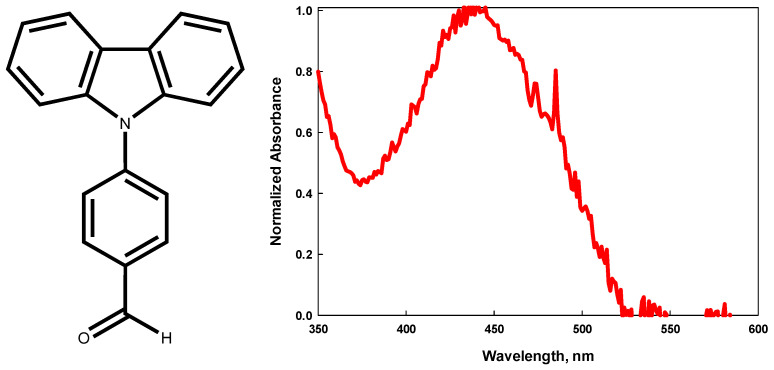
UV/vis spectra of ITO slides after electrolysis of chloroform solution of N-(4-formylphenyl) carbazole for 100 s at 2.0 V.

The UV/vis spectrum, Figure 7, shows an absorption peak at 440 nm, identical in shape and peak absorption to the peaks observed after electrolysis of **BDP1**, **BDP2**, and **BDP3** from Figure 6. It is logical to conclude that the absorption bands at 440 nm observed for **BDP1**-**BDP3** ITO-coated slides result from polycarbazole. In the case of the ITO-**BDP1** slide, the absorption band at 530 nm is the result of surface-confined **BDP1** dye, consistent with the solution absorption spectrum of **BDP1**. In contrast, the ITO-**BDP2** and ITO-**BDP3** electrocoated slides do not show any evidence for **BDP2** and **BDP3** which should show absorption bands near 600 and 640 nm, respectively. One possibility is that the more highly conjugated BDP dyes (e.g., naphthyl and fluoranthene pyrrole units) may undergo electrooxidation at the potential applied to oxidize the carbazole moieties, resulting in the decomposition of the BDP structure.

### 2.4. Investigation of a Pyrene-Substituted Isoquinol BDP Dye

**Figure 8:**
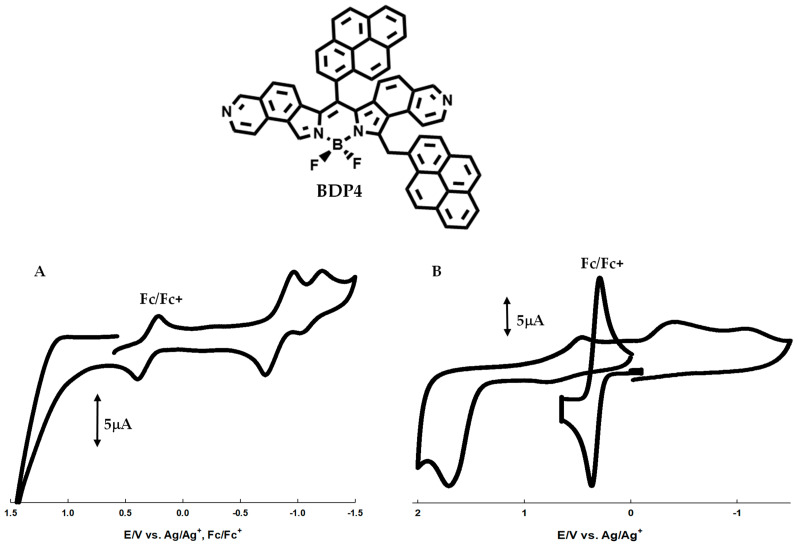
Structure of **BDP4**. (**A**) Cyclic voltammogram of a mM solution of **BDP4** in 0.1 M TBAPF_6_ in chloroform with a glassy carbon working electrode and a nonaqueous Ag/Ag^+^ reference electrode. (**B**) Cyclic voltammogram of coated glassy carbon electrode in 0.1 M TBAPF_6_ with a nonaqueous Ag/Ag^+^ reference electrode overlayed with Fc/Fc^+^ redox couple.

While **BDP1** showed the desired ability to polymerize onto ITO slides maintaining the BDP photo-properties, this was not the case with **BDP2** and **BDP3** due to a proposed breakdown of the BDP-core upon electrooxidation. In a previous study, this laboratory has reported a series of isoquinol-based BDP dyes [45]. In particular, one of these dyes showed evidence of film formation on glassy carbon electrodes upon anodic scanning in chloroform solutions. The structure of this BDP dye (**BDP4**), illustrated in Figure 8, incorporates highly conjugated pyrene moieties. Polypyrene resulting from anodic electrochemistry has been well documented [46,47,48]. The cyclic voltammetry of **BDP4** in chloroform, Figure 8A, shows that, when scanned in the anodic direction, the current begins to go off-scale. Upon scanning between 0.0 V and 1.5 V vs. the Ag/Ag^+^ reference electrode for several cycles, the glassy carbon electrode was removed from the **BDP4**/TBAP_6_ chloroform, rinsed with chloroform, placed in a 0.1 M TBAPF_6_ chloroform solution and cycled between 2.0 V and −1.5 V vs. the Ag/Ag^+^ reference electrode, Figure 8B. The coated glassy carbon electrode displays an irreversible oxidation at 1.70 V followed by two irreversible reduction waves with the most pronounced at −0.40 V vs. the Ag/Ag^+^ reference electrode.

**Figure 9:**
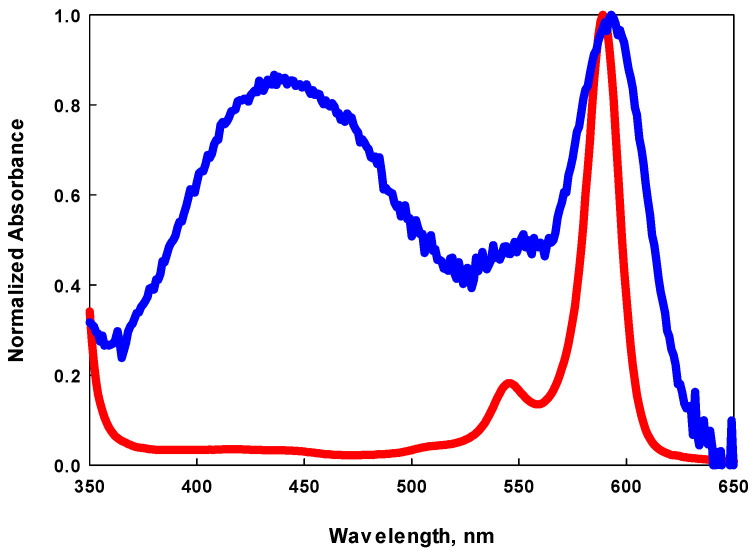
Electronic spectrum of an ITO glass slide electro-coated with **BDP4**, blue line and the spectrum of **BDP4** in chloroform solution, red line.

From these observations, mM solutions of **BDP4** in 0.1 M TBAPF_6_ chloroform containing a square ITO-coated glass slide as working electrode, platinum flag as auxiliary, and a non-aqueous Ag/Ag^+^ reference electrode were electrolyzed at 1.5 V for 100 s. The ITO slide was removed, rinsed with chloroform, and air-dried. The UV/vis spectrum of the **BDP4** coated ITO slide shows a broad absorption band at 440 nm and a sharper lower energy absorption band at approximately 600 nm, Figure 9 blue line. The absorption spectrum of a chloroform solution of **BDP4** is overlayed, Figure 9 red line, suggesting that the anodic film formation on ITO slides maintain absorption properties and, by extension, the structure of the dye.

**Figure 10:**
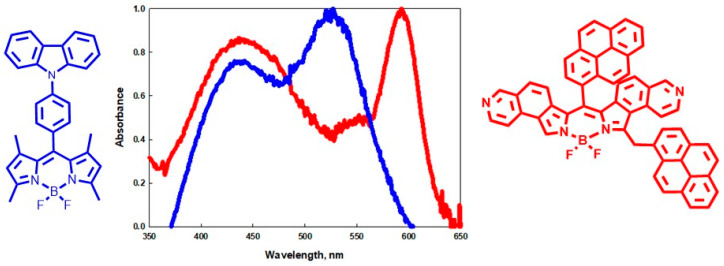
Overlayed normalized spectra of **BDP1** (blue) and **BDP4** (red) electropolymerized on ITO glass slides.

For comparison, an overlay of the spectrum of electorpolymerized BDP1 on an ITO slide (Figure 10 blue) and the electropolymerized BDP4 on an ITO slide (Figure 10 red) illustrates the region of the electromagnetic spectrum which can be covered by these films.

The objective of this study was to explore whether BDP dyes affixed with redox-active substituents could be electro-coated onto ITO slides without destroying the photophysical structure of the dyes. Solution cyclic voltammetry of the dyes revealed film formation on glassy carbon electrodes. However, when electropolymerized onto ITO slides, **BDP2** and **BDP3** clearly form films but lose their associated BDP absorption peaks. A potential explanation may be due to the ability of the extended aromatic pyrroles of **BDP2** and **BDP3** (i.e., naphthyl or fluoranthene) to undergo an oxidative process within the same potential window of the oxidation of the carbazole moiety. This leads to film formation but may also result in decomposition of the BDP-core. This may explain the differences observed for the solution cyclic voltammograms of **BDP1**, **BDP2**, and **BDP3** (Figure 3). For **BDP1**, the reduction of the dipyrrin core in the cathodic region, a one-electron process, shows approximately 1/3 the current intensity of the oxidation of the carbazole, a three-electron process, in the anodic region. On the other hand, the current intensity of the oxidative process observed for **BDP2** and **BDP3** is considerably greater than the cathodic redox process, suggesting something other than the carbazole is being oxidized. Our contention is that the additional oxidative process is due to the naphtyl (**BDP2**) and fluoranthene (**BDP3**) dipyrrin core ultimately leading to decomposition of the BDP dyes.

In contrast, ITO glass slides anodically coated with **BDP1** show a distinct BDP-like absorption band similar in shape and energy to that observed for **BDP1** in solution, Figure 2 red and Figure 10 blue, respectively. A similar process is observed for **BDP4** coated ITO glass slides (Figure 10 red) resulting from anodic oxidation of the pyrene moieties. The ITO-coated slides are stable, exhibiting similar spectroscopic properties for several days. It is apparent that while this method shows promise for affixing BDP dyes to ITO slides, judicious choice of the redox-active meso-substituents is needed to ensure that the BDP-core is unaffected by the oxidative process.

## 3. Materials and Methods

### 3.1. General Procedures

All chemicals were reagent grade and used without further purification. Naphtha[1,2-c]pyrrole, fluorantho[2,3-c]pyrrole [49] and **BDP4** [45] were synthesized as previously described. Chromatography was performed on a Teledyne Combiflash Rf^+^ equipped with UV detection. High-resolution mass spectral analysis was performed at the Mass Spectrometry and Proteomics facility at the Ohio State University. ^1^H NMR spectra were recorded on a Bruker 400 MHz NMR spectrophotometer at 298 K (Bruker, Billerica, MA, USA).

### 3.2. Spectroscopic Measurements

Electronic absorption spectra were recorded at room temperature using an HP8453 photodiode array spectrophotometer with 2 nm resolution. All spectra were recorded at 298 K. Room temperature luminescence spectra in a 1 cm quartz spectrophotometer fluorescence cell (Starna Cells, Atascadero, CA, USA) in CHCl_3_ were run on a Cary Eclipse fluorescence spectrophotometer. Luminescence quantum yields were determined at room temperature in HPLC-grade CHCl_3_ relative to Rhodamine 6G as the reference (ϕ_fl_ = 0.95, in ethanol) [50]. The quantum yields were obtained using the following equation:

ϕ_s_ = ϕ_r_[A_r_η_s_^2^D_s_/A_s_η_r_^2^D_r_] (1)

where “s” and “r” indicate the sample and reference, respectively, A is the absorbance at the excitation wavelength, η is the average refractive index of the solution, and D is the integrated area under the emission spectrum.

### 3.3. Electrochemical Measurements

Cyclic voltammograms were recorded under a nitrogen atmosphere using a one-compartment, three-electrode cell, CH-Instruments, equipped with a platinum wire auxiliary electrode. The working electrode was a 2.0 mm diameter glassy carbon disk from CH-Instruments, which was polished first using 0.30 mm followed by 0.05 mm alumina polish (Buehler, Lake Bluff, IL, USA) and then sonicated for 10 s prior to use. Potentials were referenced to a Ag/Ag^+^ non-aqueous electrode, CH-Instruments. The supporting electrolyte was 0.1 M tetrabutylammonium hexafluorophosphate (Bu_4_NPF_6_) and the measurements were made in dry CHCl_3_. Bulk electrolysis of mM CHCl_3_ solutions of the BDP dyes was performed on 2.54 cm × 2.54 cm indium tin oxide-coated square glass slides with 70–100-ohm surface resistivity (Sigma-Aldrich, St. Louis, MO, USA). The auxiliary electrode was a square platinum flag and the reference was a nonaqueous Ag/Ag^+^ electrode (CH-Instruments, Austin, TX, USA).

### 3.4. Synthesis

**BDP1** was synthesized via a variation on the previous reports using a mechanochemical technique [22]. 2,4-dimethyl pyrrole (0.25 mL, 2.4 mmol and N-(4-formylphenyl)carbazole (325 mg, 1.20 mmol) were placed in a mortar with approximately 3 mL of DCM. Approximately 10 drops of trifluoroacetic acid were added and the mixture was ground for 1 min. At this point, 454 mg (1.90 mmol) of p-chloranil was added and the mixture was ground for an additional minute. Using a syringe, 0.50 mL of triethylamine followed by 0.50 mL of boron trifluoro etherate was added while the mixture was continuously ground for another 2 min. The dark mixture was dissolved in DCM and chromatographed on silica gel using DCM with increasing amounts of methanol. Orange powder (164 mg, 28%), ^1^H NMR (400 MHz, CDCl_3_) δ 8.18 (d, *J* = 7.7 Hz, 1H), 7.72 (d, *J* = 7.8 Hz, 1H), 6.26 (s, 2H), 2.53 (s, 6H), 1.47 (s, 6H). HRMS Calcd. for C_31_H_26_N_3_BF_2_ [M^+^] 489.21878 found 489.21783.

**BDP2** and **BDP3** synthesis: To a 25 mL round-bottom flask. 50.0 mg (0.30 mmol) of naphtha [1,2-c]pyrrole or (0.21 mmol) of fluorantho[2,3-c]pyrrole and a molar equivalent of N-(4-formylphenyl)carbazole were dissolved in a minimum (ca. 1 mL) of chloroform. Under reduced pressure at 70–75 °C, the solvent was removed, giving a dark purple or greenish paste, respectively. The round-bottom flask was placed in an oil bath set at 80 °C under a nitrogen purge and approximately 5 mL of dry toluene was added followed by 250 μL of triethylamine and 300 mL of boron trifluoroetherate. The reaction mixture was stirred at 80 °C for 20 min, at which point the solution was chromatographed on silica gel with hexanes and increasing amounts of chloroform. The fluorescent products were dried, resulting in 31.5 mg (0.050 mmol, 16.5% yield) of **BDP2** and 23.2 mg (0.030 mmol, 10.0% yield) of **BDP3**.

**BDP2.**^1^H NMR (400 MHz, CDCl_3_) δ 8.88 (d, J = 21.9 Hz, 2H), 8.24 (t, J = 11.0 Hz, 2H), 8.11 (d, J = 7.7 Hz, 2H), 8.04–7.91 (m, 2H), 7.81 (dt, J = 17.0, 8.3 Hz, 2H), 7.69 (dd, J = 12.9, 5.8 Hz, 2H), 7.60 (d, J = 8.1 Hz, 3H), 7.51 (d, J = 8.7 Hz, 3H), 7.46–7.40 (m, 3H), 7.37 (d, J = 5.8 Hz, 3H), 6.59 (d, J = 8.9 Hz, 1H), 6.48 (d, J = 9.0 Hz, 1H). HRMS Calcd. for C_43_H_26_N_3_BF_2_ [M^+^] 633.21878 found 633.21890.

**BDP3.** ^1^H NMR (400 MHz, CDCl_3_) δ 8.83 (s,1H), 8.39–8.28 (m, 3H), 8.18 (s, 1H), 8.06 (dd, *J* = 21.8, 11.0 Hz, 5H), 7.99–7.79 (m, 5H), 7.78–7.68 (m, 2H), 7.69–7.54 (m, 6H), 7.39 (d, *J* = 8.8 Hz, 2H), 7.27–7.16 (m, 3H), 7.06 (s, 1H), 6.97 (s, 1H). HRMS Calcd. for C_55_H_30_N_3_BF_2_ [M^+^] 781.25008 found 781.25000.

## 4. Conclusions

A series of four BDP dyes with varying degrees of aromaticity affixed with redox-active meso-substituents have been synthesized. Solution phase spectroscopic and electrochemical properties reveal the expected HOMO-LUMO gaps associated with increasing aromaticity of the BDP-pyrroles. The carbazole substituents of **BDP1**, **BDP2**, and **BDP3** undergo oxidative redox processes, resulting in film formation on glassy carbon electrodes. Of these three dyes, only **BDP1** shows the ability to maintain the BDP-core spectroscopic properties upon oxidative film formation on ITO glass slides. Utilizing a pyrene-substituted isoquinol BDP dye (**BDP4**) shows the ability of anodic film formation with π-extended BDP dyes. Further studies are underway to determine the best meso-substituents (e.g., 4-hydroxyphenyl, 3,4-dihydroxyphenyl, and aniline) needed to allow for oxidative film formation without destruction of the BDP-core. In addition, future work will include a study of layering ITO slides with BDP-dyes with varying HOMO-LUMO gaps; ultimately, these slides will be tested as anodes in DSSCs.

## Data Availability

The data is contained within the article.
